# Natural language processing system for rapid detection and intervention of mental health crisis chat messages

**DOI:** 10.1038/s41746-023-00951-3

**Published:** 2023-11-21

**Authors:** Akshay Swaminathan, Iván López, Rafael Antonio Garcia Mar, Tyler Heist, Tom McClintock, Kaitlin Caoili, Madeline Grace, Matthew Rubashkin, Michael N. Boggs, Jonathan H. Chen, Olivier Gevaert, David Mou, Matthew K. Nock

**Affiliations:** 1 Cerebral Inc, Claymont, DE USA; 2 Stanford University School of Medicine, Stanford, CA USA; 3 Universidad de Sonora, Mexico, NA USA; 4 University of Arizona, Tucson, AZ USA; 5 Stanford Center for Biomedical Informatics Research, Division of Hospital Medicine, Clinical Excellence Research Center, Department of Medicine, Stanford, CA USA; 6 Stanford Center for Biomedical Informatics Research (BMIR), Department of Medicine and Department of Biomedical Data Science, Stanford, CA USA; 7 Massachusetts General Hospital Department of Psychiatry, Boston, MA USA; 8 Harvard University, Department of Psychology, Cambridge, MA USA

**Keywords:** Health services, Population screening

## Abstract

Patients experiencing mental health crises often seek help through messaging-based platforms, but may face long wait times due to limited message triage capacity. Here we build and deploy a machine-learning-enabled system to improve response times to crisis messages in a large, national telehealth provider network. We train a two-stage natural language processing (NLP) system with key word filtering followed by logistic regression on 721 electronic medical record chat messages, of which 32% are potential crises (suicidal/homicidal ideation, domestic violence, or non-suicidal self-injury). Model performance is evaluated on a retrospective test set (4/1/21–4/1/22, *N* = 481) and a prospective test set (10/1/22–10/31/22, *N* = 102,471). In the retrospective test set, the model has an AUC of 0.82 (95% CI: 0.78–0.86), sensitivity of 0.99 (95% CI: 0.96–1.00), and PPV of 0.35 (95% CI: 0.309–0.4). In the prospective test set, the model has an AUC of 0.98 (95% CI: 0.966–0.984), sensitivity of 0.98 (95% CI: 0.96–0.99), and PPV of 0.66 (95% CI: 0.626–0.692). The daily median time from message receipt to crisis specialist triage ranges from 8 to 13 min, compared to 9 h before the deployment of the system. We demonstrate that a NLP-based machine learning model can reliably identify potential crisis chat messages in a telehealth setting. Our system integrates into existing clinical workflows, suggesting that with appropriate training, humans can successfully leverage ML systems to facilitate triage of crisis messages.

## Introduction

Between 2000–2020, rates of suicide increased worldwide^1,2^ and by over 30% in the United States^3^. In 2020 alone, 1.2 million Americans attempted suicide and nearly 46,000 died by suicide^3,4^. Patients who are suicidal often seek help via technological platforms such as crisis hotlines, text lines, or online chat lines. In concordance with the steadily growing rates of suicide and suicidal ideation over the years, crisis hotlines have been experiencing an increased volume of callers and messagers. For example, the NAMI HelpLine saw nearly a 60% increase in help-seekers between 2019 to 2021, and despite tripling their staff, the HelpLine dropped call rate remained as high as 25%^5^.

A large problem that chat and instant messaging-based platforms face is capacity and triage. When help-seekers outnumber available responders, responders are unable to effectively respond to the plethora of incoming messages. In 2020, the National Suicide Prevention Lifeline was only able to respond to approximately 30% and 56% of incoming chats and text messages, respectively^6^.

Furthermore, many platforms use a first-come-first-serve approach^7^, where messages are placed in a queue in the order that they are received, allowing high-risk messages to be buried beneath less urgent messages. It is imperative that technology platforms receiving crisis messages be able to effectively distinguish between urgent and non-urgent messages.

Nascent research has demonstrated that machine learning (ML) can be applied to automate triage of crisis messages^8–11^. One model built by Xu et al., achieved high positive predictive value and sensitivity for non-crises cases (0.984 and 0.942, respectively) and for crises-cases achieved precision and recall of 0.649 and 0.870, respectively^8^. Crisis Text Line R&D built a system that uses two binary classification models to quickly identify messages that indicate suicidal risk and ongoing self-harm (recall = 0.89)^7^.

To improve response times to patients in crisis, we developed Crisis Message Detector-1 (CMD-1), a natural language processing (NLP) system to detect potential crisis messages sent by patients of a large, national tele-mental health platform that serves over 200k patients. Patients can send chat messages to their clinicians via a mobile web platform, and many use the chat feature to reach out for help during an acute behavioral health crisis. CMD-1 was designed to support the crisis response team, whose role is to support patients experiencing crises, including suicidal/homicidal ideation, domestic violence, or non-suicidal self-injury. The system was used to surface concerning messages to the crisis response team with the aim of decreasing response times to patients in crisis. Importantly, CMD-1 aided but never replaced human review of patient chat messages—all surfaced messages were reviewed by a human prior to patient intervention, and any messages not surfaced by the model were reviewed by a human as part of the typical crisis response workflow. We describe how the crisis specialist team used CMD-1 as part of their clinical workflows and report the results of a retrospective validation and large prospective validation (over 120,000 messages).

## Results

### Message and patient characteristics

The training set included 721 messages (32% true crisis events) sent between April 1, 2021 and April 1, 2022 from 563 distinct patients (Table 1). There was a median of 1 (95% CI: 1–1) message sent per patient, and the median number of characters per message in the training set was 201 (95% CI: 181–221). Of the 563 distinct patients, 74% were female, 65% were aged 25 to 45, 29% had a diagnosis of generalized anxiety disorder with or without other comorbidities, and 32% had been in treatment between two to four weeks when their first message in the training set was sent.

**Table 1:** Message characteristics and patient demographic and clinical characteristics for the training set (*n* = 721); retrospective test set (*n* = 481); and prospective test set, separated by messages that passed through the crisis terms filter and were presented to the prediction model (*n* = 9795), and those that did not pass through the crisis terms filter and were automatically classified as non-crises (*n* = 92,676).

Metric	Training set	Retrospective test set	Prospective test set—passed through filter	Prospective test set—did not pass through filter
Message-level attributes				
Total N distinct messages	721	481	9795	92,676
*N* true crisis events (%)	225 (31.1%)	158 (32.8%)	529 (5.4%)	28 (0.03%)
Median N messages per patient (95% CI)	1 (95% CI: 1–1)	1 (95% CI: 1–1)	1 (95% CI: 1–1)	2 (95% CI: 2–2)
Date range of messages included	4/1/21–4/1/22	4/1/21–4/1/22	10/1/22–10/31/22	10/1/22–10/31/22
Median N characters per message (95% CI)	201 (95% CI: 181–221)	204 (95% CI: 184–219)	170 (95% CI: 167–173)	83 (95% CI: 82–84)
Patient-level attributes				
Total N distinct patients	563	384	6973	31,225
Gender				
Female	419 (74%)	279 (73%)	4832 (69%)	21,157 (68%)
Male	135 (24%)	96 (25%)	1899 (27%)	8790 (28%)
Other	9 (1.6%)	9 (2.3%)	242 (3.5%)	1278 (4.1%)
Age (years) at date of first message sent				
18–25	129 (23%)	81 (21%)	1161 (17%)	5283 (17%)
25–45	367 (65%)	248 (65%)	5051 (72%)	22,765 (73%)
45–60	55 (9.8%)	49 (13%)	664 (9.5%)	2779 (8.9%)
>60	12 (2.1%)	6 (1.6%)	97 (1.4%)	398 (1.3%)
Mental health diagnosis at the time of first message sent				
Generalized anxiety disorder (+other comorbidities)	177 (31%)	108 (28%)	2403 (34%)	10,405 (33%)
Major depressive disorder (+other comorbidities excluding GAD)	160 (28%)	97 (25%)	1988 (29%)	8775 (28%)
Bipolar disorder (+other comorbidities excluding GAD and MDD)	41 (7.3%)	26 (6.8%)	567 (8.1%)	2170 (6.9%)
Other	88 (16%)	69 (18%)	1757 (25%)	8604 (28%)
No diagnosis	97 (17%)	84 (22%)	258 (3.7%)	1271 (4.1%)
Days from treatment initiation to first message sent				
<2 weeks	112 (20%)	78 (20%)	240 (3.4%)	1681 (5.4%)
2–4 weeks	178 (32%)	112 (29%)	3589 (51%)	23,805 (76%)
4–8 weeks	83 (15%)	54 (14%)	152 (2.2%)	961 (3.1%)
8–12 weeks	82 (15%)	67 (17%)	226 (3.2%)	1591 (5.1%)
>12 weeks	67 (12%)	31 (8.1%)	237 (3.4%)	1423 (4.6%)
Unknown	41 (7.3%)	42 (11%)	2529 (36%)	1764 (5.6%)

The retrospective test set included 481 messages (32% true crisis events) sent between April 1, 2021 and April 1, 2022 from 384 distinct patients. There was a median of 1 (95% CI: 1–1) message sent per patient, and the median number of characters per message in the training set was 204 (95% CI: 184–219). Of the 384 distinct patients, 73% were female, 65% were aged 25 to 45, 27% had a diagnosis of generalized anxiety disorder with or without other comorbidities, and 29% had been in treatment between two to four weeks when their first message in the training set was sent.

The prospective test set included 102,471 messages (0.55% true crisis events) sent between October 1, 2022 and October 31, 2022 from 32,803 distinct patients (SI Table 1). Of these, 9,795 (5.4% events) passed through the crisis terms filter and 92,676 (0.03% events) did not. There was a median of 2 (95% CI: 2–2) messages sent per patient, and the median number of characters per message in the training set was 92 (95% CI: 91–92). Of the 32,803 distinct patients, 68% were female, 73% were aged 25 to 45, 34% had a diagnosis of generalized anxiety disorder with or without other comorbidities, and 79% had been in treatment between two to four weeks when their first message in the training set was sent.

Overall, the training set was similar to the retrospective test set, but contained a smaller proportion of males (25% vs. 28%), greater proportion of patients aged 18–25 (23% vs. 17%), smaller proportion of patients with generalized anxiety disorder (29% vs. 34%), and smaller proportion of patients who were in treatment between 2–4 weeks before their first message was sent (32% vs. 79%).

Compared to patients in the retrospective test set, patients in the prospective test set were more likely to be male (28% vs. 25%), to have generalized anxiety disorder (34% vs. 28%), and to have been in treatment for 2–4 weeks (79% vs. 29%). When comparing messages in the prospective test set that passed through the crisis terms filter to those that did not, messages that passed through the crisis terms filter were longer (170 characters [95% CI: 167–173] vs. 83 [95% CI: 82–84]).

### CMD-1 performance

On the retrospective test set, the model had an AUC of 0.82 (95% CI: 0.784–0.862), sensitivity of 0.99 (95% CI: 0.955–0.998), specificity of 0.12 (95% CI: 0.085–0.158), PPV of 0.35 (95% CI: 0.309–0.4), and NPV of 0.95 (95% CI: 0.831–0.994) (Table 2). The calibration slope was 0.48 (*p* = <0.001) and the calibration intercept was 0.43 (*p* < 0.001). The calibration curve and plot showed reasonable concordance between actual and predicted event probabilities (SI Figs. 1 and 2). The features selected by the model were reasonably associated with the crisis outcome (SI Table 3).

**Table 2:** Model performance for the validation set (*n* = 481) and prospective test set (*n* = 102,471).

Metric	Retrospective test set	Prospective test set
AUC	0.82 (95% CI: 0.78–0.86)	0.975 (95% CI: 0.966–0.984)
Probability threshold for binary classification	0.01	0.01
Sensitivity	0.99 (95% CI: 0.955–0.998)	0.975 (95% CI: 0.958–0.986)
Specificity	0.12 (95% CI: 0.085–0.158)	0.970 (95% CI: 0.966–0.973)
PPV	0.35 (95% CI: 0.309-0.4)	0.66 (95% CI: 0.626–0.692)
NPV	0.95 (95% CI: 0.831–0.994)	0.99 (95% CI: 0.997–0.999)
Calibration slope	0.48 (*p* < 0.001)	0.96 (*p* < 0.001)
Calibration intercept	0.43 (*p* < 0.001)	–1.43 (*p* < 0.001)

For measures of classification accuracy, the indicated probability threshold was applied. The Wald test was used to calculate *p*-values for calibration slope and intercept.

On the prospective test set, the crisis term filter had a sensitivity of 0.993 (95% CI: 0.982–0.998), specificity of 0.910 (95% CI: 0.908–0.911), PPV of 0.0567 (95% CI: 0.052–0.062), and NPV of 0.99996 (95% CI: 95% CI: 0.999–1.00) (SI Table 2). The logistic regression model showed stronger discrimination, with an AUC of 0.99 (95% CI: 0.987–0.991), sensitivity of 0.97 (95% CI: 0.95–0.981), specificity of 0.97 (95% CI: 0.966–0.973), PPV of 0.66 (95% CI: 0.626–0.692), and NPV of 0.99 (95% CI: 0.997–0.999). The calibration slope was 0.96 (*p* = <0.001) and the calibration intercept was –1.43 (*p* < 0.001). The calibration curve and plot showed reasonable concordance between actual and predicted event probabilities when the predicted probabilities were greater than 0.5 or less than 0.1, and overestimation of event probabilities when the predicted probabilities were between 0.1 and 0.5 (SI Figs. 1 and 2).

**Fig. 1:**
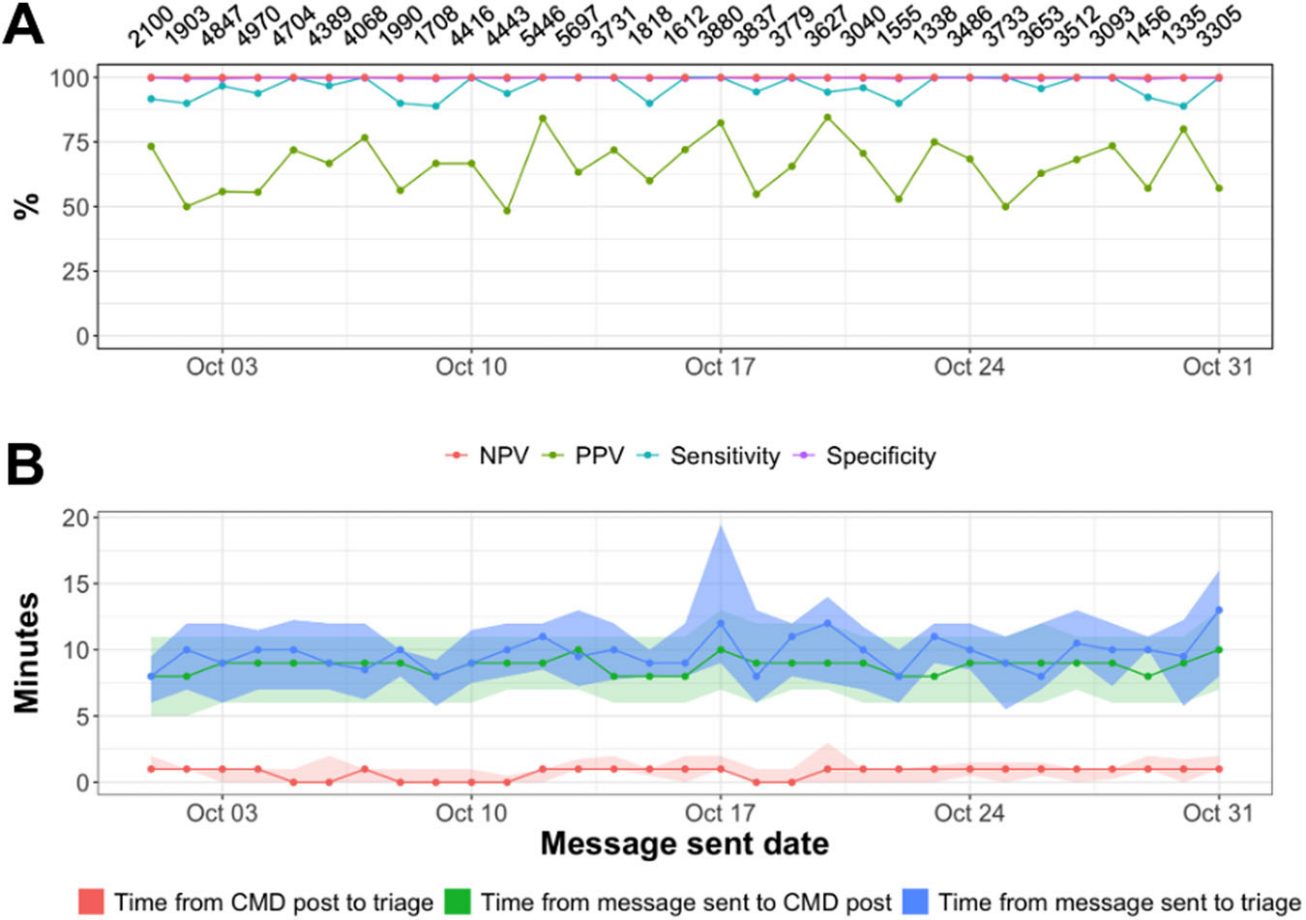
Model performance and response time metrics for the prospective test set (*n* = 120,471 total messages from 10/1/22–10/31/22). **A** Discrimination metrics by day along with the total number of messages sent per day. **B** Response time statistics across three sequential time points: (1) message sent by patient, (2) CMD-1 post to crisis specialist team, and (3) crisis specialist triage. Median (solid lines) and 25th–75th percentile range (shaded) are shown.

For the prospective test set (10/1/22–10/31-22), model performance was measured daily. Sensitivity ranged from 0.89 to 1.00, specificity ranged from 0.994 to 0.999, PPV ranged from 0.48 to 0.85, and NPV ranged from 0.999 to 1.000 (Fig. 1).

Among patients aged 18–21 in the prospective test set, the model had an AUC of 0.98 (95% CI: 0.9756–0.9936), sensitivity of 0.976 (95% CI: 0.871–0.999), specificity of 0.965 (95% CI: 0.944–0.979), PPV of 0.702 (95% CI: 0.566–0.816), and NPV of 0.998 (95% CI: 0.988–1). The calibration slope was 1.21 (*p* = <0.001) and the calibration intercept was –1.34 (*p* < 0.001) (Table 3).

**Table 3:** Model performance for patients aged 18–21 within the prospective test set.

Metric	Value
Total N distinct messages sent	5579
Total N distinct messages passing terms filter	525
N true crisis events (%)	0.72%
AUC	0.98 (95% CI: 0.9756–0.9936)
Probability threshold for binary classification	0.01
Sensitivity	0.98 (95% CI: 0.871–0.999)
Specificity	0.97 (95% CI: 0.944–0.979)
PPV	0.70 (95% CI: 0.566–0.816)
NPV	1.00 (95% CI: 0.988-1)
Calibration slope	1.21 (*p* < 0.001)
Calibration intercept	–1.34 (*p* < 0.001)

The Wald test was used to calculate *p*-values for calibration slope and intercept.

For measures of classification accuracy, the probability threshold of 0.01 was applied.

Failure analysis of 17 false negatives (SI Table 4) revealed that the 4 messages that did not pass through the crisis terms list contained phrases not included in the terms list (e.g., “ER,” “tired of being alive,” “not exist,” “not feeling okay”).

### Response times

**Fig. 2:**
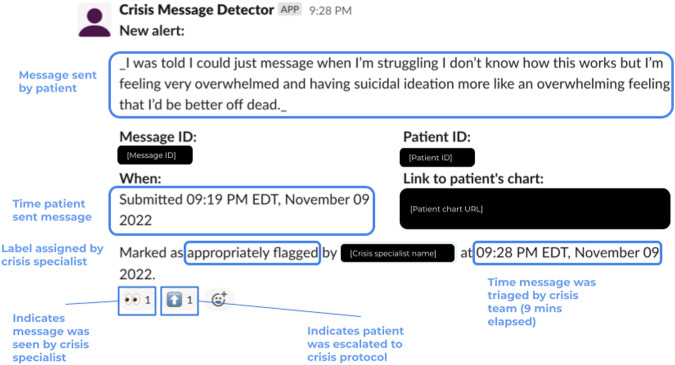
An example Slack post made by the CMD-1 alerter to crisis specialists. The post contains the text of the predicted crisis message, unique message and patient identifiers, a link to the patient’s EMR chart, the time the message was sent by the patient, the label (“appropriately flagged” or “inappropriately flagged”) recorded by the crisis specialist, and emoticons used by crisis specialists.

From October 1 to October 31, the daily median time from message sent to CMD-1 post ranged from 8 to 11 min (average IQR 5.1 min), the daily median time from CMD-1 post to crisis specialist triage ranged from 0 to 1 min (average IQR 1.1 min), and the daily median time from message sent to crisis specialist triage ranged from 8 to 13 min (average IQR 4.7 min) (Fig. 2). Prior to deploying CMD-1, internal analysis showed that response times to chat messages were over 9 h on average.

## Discussion

While crisis helplines provide much needed support to individuals experiencing mental health emergencies, they can be limited by human capacity^12^ and are typically not integrated within healthcare systems. With increasing volumes of requests for help^5^ that are triaged on a first-come-first-serve basis, time-sensitive crisis messages from high-risk individuals may wait in a queue behind less urgent messages. We built CMD-1, an NLP-enabled system that detects and surfaces crisis messages to enable faster triage from a crisis response team. We deployed CMD-1 within a large, national telehealth provider platform and performed a prospective validation on over 120k messages coming from over 30k distinct patients. We found that CMD-1 was able to detect high-risk messages with high accuracy (97.5% sensitivity and 97.0% specificity) and enabled crisis specialists to triage crisis messages within 10 min (median) of message receipt.

The speed at which a high-risk individual in crisis is contacted is especially important because immediate intervention can ultimately divert them away from a suicide attempt (Kelly et al., 2008; McClatchey et al., 2019). CMD-1 reduced response times to crisis chat messages by nearly two orders of magnitude, from over 9 h on average to 9 min (median). This supports the findings of previous work using ML to enhance a clinical team’s capability to satisfactorily address the crises of high-risk individuals (Crisis Text Line, and Xu et al., 2021). For example, Crisis Text Line used a machine learning model to reduce wait times for high risk texters from 8 min (median, 75th percentile: 35 min) down to 3 min (median, 75th percentile: 11 min). One key advantage of CMD-1 is that it was integrated within the patient’s clinical workflow, allowing clinicians to be notified of patient crises as they happen. Indeed, we have shown that notifying clinicians of patient crises can lead to more timely follow-up care^13^. Crisis response systems that are integrated within provider organizations have the potential to impact key aspects of patient care. Future work might investigate the impact of CMD-1 and response times on downstream health outcomes such as hospitalization or ED utilization.

Despite growing interest in ML applications in healthcare in recent years, ML models are rarely deployed in health systems, with most studies presenting model development without translation to clinical care^14^. Deploying a ML model into clinical workflows requires technical considerations (e.g., model accuracy), operational considerations (e.g., model interpretability, user experience), and technological infrastructure (e.g., data storage capabilities, ability to generate predictions in near-real time)^15,16^. In developing CMD-1, a cross-functional team of clinicians worked together to address these considerations. Clinicians and data scientists collaborated to define a meaningful outcome and select an appropriate probability threshold for classification. Clinicians and data engineers collaborated to design a simple, effective user interface for the CMD-1 Slack posts. Data scientists and data engineers collaborated to embed the ML model within a robust data infrastructure that enabled near real-time predictions and data capture.

The superior performance of CMD-1 in the prospective test set compared to the retrospective test set warranted further investigation. We hypothesized that the higher AUC (0.98 vs. 0.82) and higher specificity (0.97 vs. 0.12) in the prospective test set compared to the retrospective test set was due to differences in both the data sampling methodology as well as class imbalance. To increase the prevalence of true crises in the training and retrospective test sets, we included all messages sent in the seven days prior to a true crisis, as recorded in a crisis event tracker. While this enabled us to enrich our training set with true crisis messages, the included non-crisis messages were not representative of non-crisis messages in the deployment setting. We hypothesized that the non-crisis messages preceding crisis events were more difficult to distinguish from crisis messages than non-crisis messages not associated with crisis events, and that this explained the lower AUC and specificity during retrospective evaluation. For example, the message “I want nothing. I used to want things, now I don’t want anything… I sense a danger”, which preceded a crisis, was likely more difficult to accurately classify as a non-crisis than the message “Thanks for helping me meet with someone,” which did not precede a crisis. The fact that non-crisis messages in the prospective test set were shorter than those in the training and retrospective test sets lends further support to this idea.

To confirm this hypothesis, we show that the median predicted probability for non-crisis messages in the retrospective test set was 0.079 [IQR: 0.026–0.170], while the median predicted probability for non-crisis messages in the prospective test set was 0.019 [IQR: 0.0027–0.069], which is much closer to the model’s decision boundary of 0.01 (SI Table 5).

In addition, the difference between retrospective and prospective test set values caused by data sampling methodology was exacerbated by the increased proportion of non-crisis messages in the prospective test set. To further confirm this hypothesis, we conducted a sensitivity analysis where we randomly down-sampled the proportion of non-crisis messages in the prospective test set so that the event rate matched that of the retrospective test set. Over 100 iterations, the median AUC on the modified prospective test set with down-sampled true negatives was 0.82 [IQR: 0.82–0.82], which nearly exactly matches the AUC of 0.82 in the retrospective test set (SI Fig. 3). This suggests that the performance of CMD-1 was comparable in both retrospective and prospective test sets, and that differences in AUC could largely be attributed to differences in the proportion of and type of non-crisis messages. Other contributing factors to the differences in model performance may include changes in the patient population between April and October 2022. For example, compared to patients in the retrospective test set, patients in the prospective test set were more likely to be male, to have generalized anxiety disorder, and to have been in treatment longer.

One limitation of our approach is our use of the crisis terms filter. As discussed above, the terms filter excluded messages from being evaluated by the NLP model if they did not contain at least one phrase from the filter. While our filter was broad and curated manually by experts, some messages that may otherwise be flagged as crises could be filtered out by the terms filter. One way to quantify this would be an ablative study—removing one term from the terms list at a time and quantify changes in our experimental outcomes. Using this, one can estimate the number of messages that would be flagged as crises after adding more terms to the filter.

Another limitation is the size of our training set and the fact that we artificially enriched the training set for true crises. As described in “Methods” section, due to the low prevalence of crisis messages (<1%), we enriched the training set and retrospective test set for crisis messages for efficiency of data labeling. The training set was 721 messages, and the prevalence of crisis messages in the prospective test set was 0.6% compared to 32% in the training and retrospective test sets. Applying a prediction model to a dataset with a different event rate than the dataset used to train the model can result in mis-calibrated predicted risks, thus impacting downstream classification^17^. Further, class imbalance corrections like random under-sampling of non-events without subsequent recalibration has been shown to lead to miscalibration. There are two reasons why these risks of miscalibration were mitigated when deploying CMD-1. First, CMD-1 is a classifier and not a continuous risk prediction model, meaning that the impact of miscalibration on model discrimination is limited to predicted risks close to the classification threshold. This is reinforced by the strong sensitivity and specificity of the model on the prospective test set. Second, deploying CMD-1 in a population where the event rate is lower than that of the training dataset would be expected to lead to inflated predicted risks and therefore more false positives, not more false negatives. This is acceptable given our 20 to 1 preference for false positives over false negatives for this use case.

The moderate PPV of CMD-1 could also be considered a limitation. With approximately four out of every 10 messages surfaced being a false positive, there is ample opportunity to improve the accuracy of CMD-1 to decrease wasted human effort of triaging false positives. Exploring the performance of other word embeddings like word2vec or other prediction functions like support vector machines, random forest, or deep learning architectures such as large language models could improve the PPV of CMD-1 without sacrificing sensitivity.

Overall, CMD-1 serves as a promising model for ML-enabled solutions to drive improvements in mental healthcare delivery. By using technology to automate a manual task, CMD-1 increases operational efficiency and reduces wait times for patients. With demand for mental health services far exceeding supply, providers must leverage technology and data to increase access to care and make the best use of available human capacity.

## Method

This study followed the Transparent reporting of a multivariable prediction model for individual prognosis or diagnosis (T RIPOD) guidelines.

### Data source

Cerebral is a national tele-mental health provider that receives thousands of patient messages daily via a HIPAA-compliant chat system available through a web and mobile application. This allows patients to connect with their care team on a variety of topics such as appointment rescheduling, medications, and more. It also serves as a form of contact for patients who are experiencing a crisis. To develop our model, we considered messages in Cerebral’s database that were sent by patients via the chat system between 04/01/2021 and 10/31/2022. Model development and validation was performed using three datasets. The training set and retrospective test set included messages sent between 04/01/2021 and 04/01/2022 (random 60:40 split between training set and retrospective test set). The prospective test set included messages sent between 10/01/2022 and 10/31/2022.

### Ethics approval

All patients provided written consent to receive telehealth services at Cerebral, which is a prerequisite to receiving care and accessing the chat system. This study was an analysis of routinely collected electronic health record data, and posed no additional risk to patients. The Stanford University IRB determined that this study does not qualify as human subjects research, and as such did not warrant further review.

### Inclusion criteria and crisis terms filter

Crisis messages were rare in the total population of messages sent from 04/01/2021 through 04/01/2022 ( < 1%). To increase the efficiency of data labeling, we excluded messages that did not contain phrases that were commonly found in crisis messages. We developed a crisis terms filter that contained 275 common words and phrases found in crisis text messages, such as “feel terrible,” “hopelessness,” and “negative thoughts” (Supplementary Information “Crisis term list”). Words in the terms filter were lemmatized to maximize sensitivity. Lemmatization groups the inflected forms of a word into one entity (e.g., “ran,” ”runs,” “run” → “run”). Messages that did not match a term in the crisis terms filter were excluded from the training set and retrospective test set. Additionally, when the machine learning model was deployed on the prospective test set, only messages that passed through the crisis terms filter were passed to the model for prediction. As more crisis messages were identified throughout the labeling process (described below) the terms filter was updated.

Starting with all 10,063,900 messages sent in the electronic medical record (EMR) chat between 04/01/2021 and 04/01/2022, we randomly sampled 200,000 for ease of data processing. We then excluded 145,737 messages sent by care team members, yielding 54,263 messages sent by patients. We then applied the crisis term filter, which excluded 50,213 messages, yielding 4050 messages that included a term associated with a crisis. We then excluded duplicate messages, yielding 3969 messages that were eligible for labeling. In general, duplicate messages tended to be shorter (e.g., “hello?,” “ok,” “yes,” “no”) than the average message length.

**Fig. 3:**
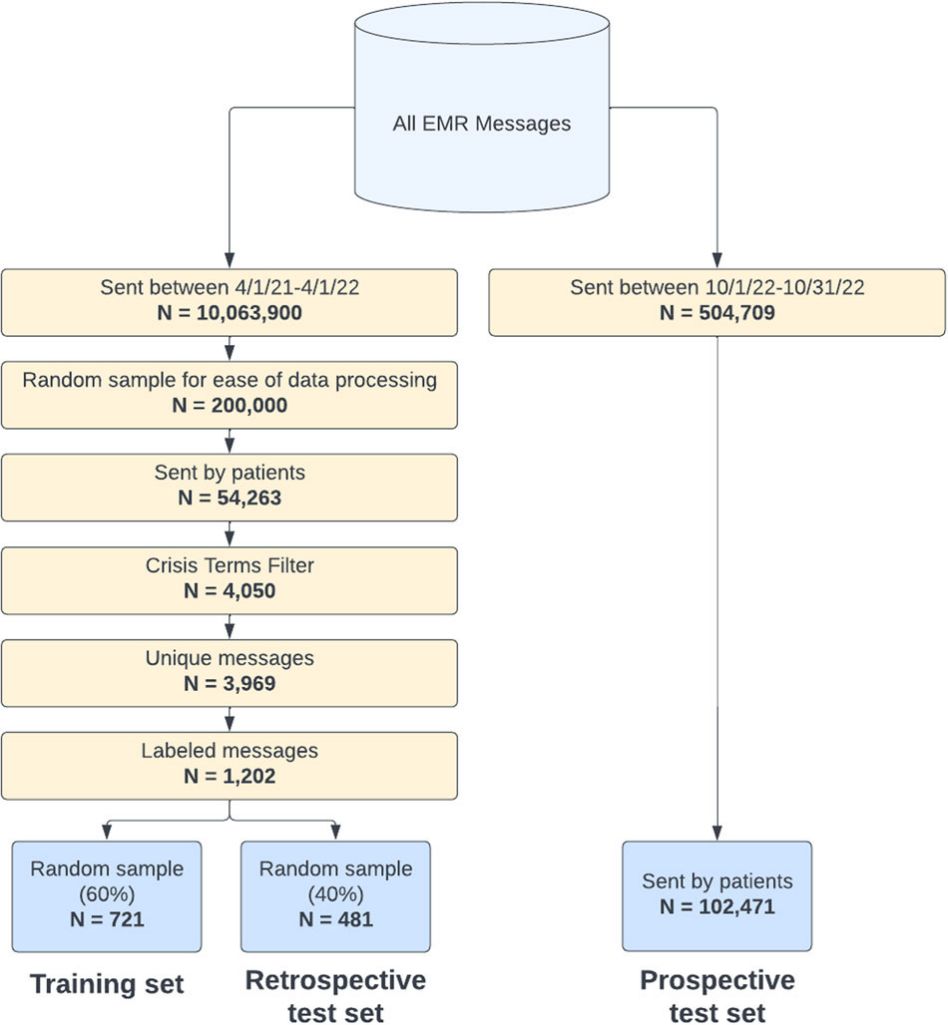
Cohort inclusion criteria. Flow diagram depicting inclusion criteria for the training set, retrospective test set, and prospective test set.

From the 3969 messages eligible for labeling, the labeled messages that made up the training and retrospective test sets were selected in two stages. First, a random sample of 596 messages sent between 04/01/2021 and 10/31/2022 were labeled. Of these only 17 (2.8%) were crisis messages. To increase the proportion of crisis messages in the labeled set, we used a tracker of patient crises maintained by the Crisis Response team. This tracker documented instances of all patient crises that were self-reported by patients and escalated to the Crisis Response team. We cross-referenced the data in this tracker with EMR chat message data to identify messages sent by patients in crisis that—we hypothesized—were more likely to indicate a crisis. For example, if the tracker mentioned that a patient reported a crisis on 5/1/21 at 5 pm ET, we labeled all chat messages sent by that patient up to seven days before 5/1/21 at 5 pm ET. Using this approach, we labeled an additional 606 messages, of which 365 (60%) were crises. Taken together, the labeled set included 596 + 606 = 1202 messages, of which 382 (32%) were true crises. Of these, 721 (60%) were randomly sampled for the training set and 481 (40%) were randomly sampled for the retrospective test set (Fig. 3). Before deploying the model on the prospective test set, the model was re-trained on all labeled data (1202 messages).

As part of prospective validation, we deployed the model between 10/01/2022 and 10/31/2022. Out of all 504,709 messages sent between those dates, we excluded messages sent by care team members, yielding 102,471 messages sent by patients. These messages comprised the prospective test set. Because the prospective validation was designed to evaluate the performance of both the crisis terms filter and the machine learning model, messages that did not pass through the crisis terms filter were not excluded from the prospective test set. Given the prospective model deployment, duplicate messages were not excluded (Fig. 3).

### Outcome

The outcome was a binary indicator of whether a message warranted further attention from a crisis specialist. A message warranted further attention if it indicated possible suicidal ideation, homicidal ideation, domestic violence, or non-suicidal self-injury.

- Suicidal ideation was defined as “thoughts about or a preoccupation with killing oneself, often as a symptom of a major depressive episode”^18^.
- Homicidal ideation was defined as “Thinking about ending or making plans to end another’s life”^19^.
- Domestic violence was defined as “a pattern of abusive behavior in any relationship that is used by one partner to gain or maintain power and control over another intimate partner… Domestic violence can be physical, sexual, emotional, economic, psychological, or technological actions or threats of actions or other patterns of coercive behavior that influence another person within an intimate partner relationship”^20^.
- Non-suicidal self-injury was defined as “The direct, deliberate destruction of one’s own body tissue in the absence of suicidal intent”^21^.

Examples of messages that would warrant further attention include: “I have so many suicidal thoughts” and “I want to end it now”. Messages that were ambiguous were considered to warrant further attention. Examples of ambiguous messages include “I need help” and “I’m so depressed”.

Data labeling on the training set and retrospective test set was performed by three labelers who were trained by crisis specialists, and data points where labelers were uncertain were reviewed by crisis specialists. Labeler inter-rater reliability calculated on 300 random messages was high, with perfect concordance on 92.8% of messages. Discrepant messages were adjudicated by crisis specialists. In total, there were 1202 labeled messages used for model development (721 training set and 481 retrospective test set).

**Fig. 4:**
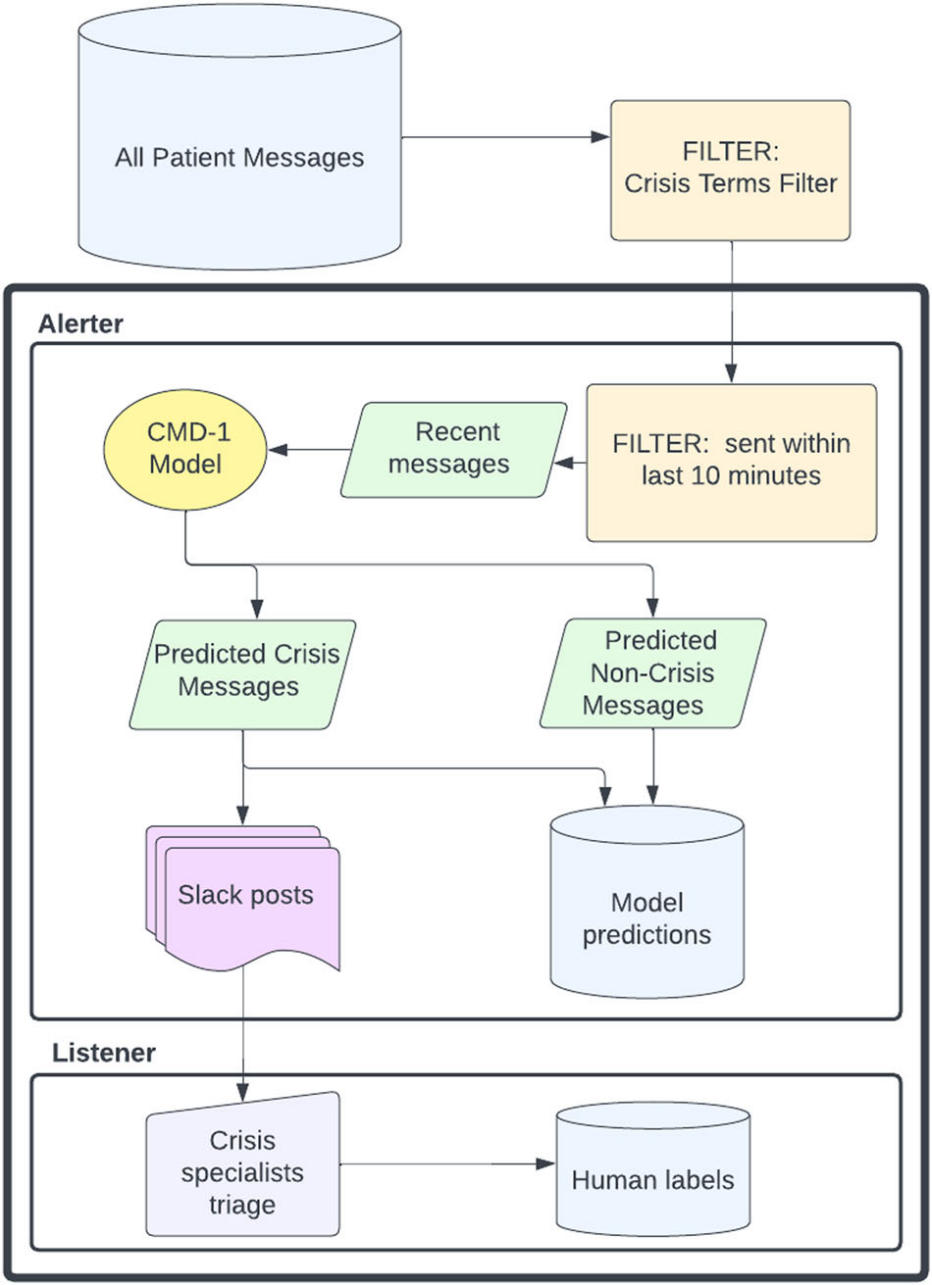
Diagram depicting the flow of patient messages through the CMD-1 system in model deployment. All patient messages first passed through a regular-expressions-based crisis terms filter. The model was applied to recent messages every 10 min, triggered by the “alerter,” a script run using AWS Lambda. Predicted crisis messages are posted by the alerter to Slack where they are reviewed and labeled by crisis specialists. The labels assigned by crisis specialists are captured in a database by the “listener,” a script run using AWS Lambda.

Data labeling on the prospective test set for true positives, false positives, and false negatives was performed by crisis specialists. For messages that the model surfaced (true positives and false positives) labels were recorded by crisis specialists using the Slack user interface of CMD-1 (described below, Fig. 4). For messages that the model did not surface (true negatives and false negatives), those that were crisis messages (false negatives) were eventually surfaced to crisis specialists through usual channels (e.g., patient chat support personnel would raise the message to a crisis specialist) and the crisis specialist would record the false negative in a spreadsheet. We expected this method of labeling false negatives to be high fidelity as all crisis messages are required by policy to be routed to the crisis specialist team. All messages that were not surfaced by CMD-1 and that were not recorded by crisis specialists as false negatives were presumed to be true negatives.

### Predictors

Machine learning modeling was performed on messages that passed through the crisis terms filter. Modeling was performed at the level of individual messages (a particular patient may have sent multiple messages). Features were derived entirely from the text of the messages. TF-IDF^22^ (term-frequency-inverse-document-frequency) scores were calculated for unigrams, bigrams, trigrams, 4-grams, and 5-grams. Briefly, TF-IDF is a method used to quantify the relative frequency of an n-gram (an n-word phrase) in a corpus of documents. For example, the TF-IDF score for the unigram “hurt” in a patient’s message report is proportional to the frequency of the word “hurt” in that patient’s pathology report (term frequency) divided by the frequency of the word “hurt” across all messages across all patients (inverse document frequency).

### Statistical analysis

#### Model development

The model was trained on messages from 04/01/2021 to 04/01/2022 (training set, 721 messages), tested retrospectively on messages from 04/01/2021 to 04/01/2022 (retrospective test set, 481 messages), and tested prospectively on message from 10/01/2022 to 10/31/2022 (prospective test set, 120,471 messages). L1-regularized logistic regression (Lasso) was used for feature selection and prediction. Hyperparameters (regularization for Lasso and sparsity for document term matrix) were tuned using 10-fold cross-validation on 60% of the training set to minimize misclassification cost (Equation (1)). Selected features were examined for sensibility and clinical meaningfulness (Supplementary Information Table 1).

1 $$cost={C}_{fp}\cdot {N}_{fp}+{C}_{fn}\cdot {N}_{fn}+{C}_{u}\cdot {N}_{u}$$

Where FP is false positive and FN is false negative.

Here, cost refers not to monetary cost, but rather the real-world utilities corresponding to FPs and FNs. In this case, the cost of an FP is the cost of incorrectly surfacing a non-crisis message (e.g., increased review burden on crisis specialists) and the cost of an FN is the cost of incorrectly missing a true crisis message (e.g., delayed intervention for a patient in distress). It is critical that the probability threshold for classification models be selected to reflect the end user’s relative tolerance for FPs vs. FNs. To this end, we surveyed six relevant stakeholders (clinicians, business leaders, data scientists) using a regret-based approach to assess the relative costs of false positives and false negatives. The survey asked the respondent to quantify how many false positives (messages incorrectly surfaced by model) they would be willing to review manually to avoid a false negative (true crisis message not surfaced by model). The survey results were used to inform a discussion among stakeholders, and ultimately a FP : FN cost ratio of 1:20 was selected, meaning false negatives were 20 times more undesirable than false positives.

The probability threshold for binary classification was selected by finding the threshold that produced the minimum misclassification cost. This is mathematically equivalent to using the threshold of 1 / (1 + 20/1) = 0.048 in a well calibrated model^23^. Messages with predicted probabilities greater than the probability threshold were labeled as crisis messages, and messages with predicted probabilities below the probability threshold were labeled as non-crisis messages.

#### Performance evaluation

Performance of the crisis terms filter in the prospective test set was calculated using sensitivity, specificity, positive predictive value (PPV), and negative predictive value (NPV). Area under the receiver operator curve (AUC), sensitivity, specificity, PPV, and NPV were used to evaluate model discrimination. 95% confidence intervals were calculated using Clopper-Pearson^24^ for sensitivity, specificity, PPV, and NPV, and for AUC using the approach developed by DeLong et al.^25^. For the prospective test set, discrimination metrics were calculated by day, by week, and for the overall test set. Calibration in the mean and measures of weak and moderate calibration (calibration slope, calibration intercept, and calibration curves) were calculated for the retrospective and prospective test sets.

As part of a failure analysis, we manually reviewed all false negatives. False negatives that did not pass through the crisis terms filter were reviewed for phrases that could be added to the crisis terms filter. False positives were not reviewed.

#### Subgroup analysis

As a subgroup analysis, we investigated model performance in the prospective test set for patients aged 18–21 years. This group represents the youngest patient population treated at Cerebral, and is of particular clinical interest for many reasons. First, young people may use different language patterns (slang, vernacular) compared to older patients, which may affect the ability of the model to recognize crisis messages for this subgroup. Second, young people have a higher risk of suicidal thinking and suicide attempts^26^, and therefore represent an important vulnerable population.

#### Analysis of response times

Since CMD-1 was designed to improve response times to patients in crisis, we considered three time points for all messages in the prospective test set. The first time point was when the message was received from the patient (henceforth referred to as the “message sent” time). The second time point was when CMD-1 surfaced the message in the Slack channel (henceforth referred to as the “CMD post” time). The third time point was when a crisis specialist marked the surfaced message as “appropriately flagged” or “inappropriately flagged”, indicating that they had reviewed the message and triaged it appropriately (henceforth referred to as the “crisis specialist triage” time). We measured time from message sent to CMD post, CMD post to crisis specialist triage, and message sent to crisis specialist triage. Summary statistics like median and interquartile range (IQR) were calculated.

### Workflow and user interface

The model was deployed to a production environment where it could receive messages in near-real time, generate predictions, and surface predictions to the crisis specialist team (Fig. 3). The model was deployed in two parts: an “alerter” and a “listener”. The alerter consisted of a script that ran every ten minutes on AWS Lambda. Within the alerter script, messages sent in the EMR over the last ten minutes were retrieved and processed with the NLP model. Messages with a high predicted probability of being a crisis were posted to slack using the slack API^27^ for the crisis response team to assess. Each post from the alerter contains information about the predicted crisis message, including the text of the message, a link to the patient’s EMR profile, and the time the message was sent (Fig. 4). The post also contains buttons that crisis specialists can use to tag the message as “appropriately flagged” if it is a true positive or “inappropriately flagged” if it is a false positive. Pushing these buttons triggered the listener, deployed inside a different AWS Lambda, which then stored these data in a database and can be used for downstream model performance analysis and retraining.

### Reporting summary

Further information on research design is available in the Nature Research Reporting Summary linked to this article.

### Supplementary information

Supplementary Information

Reporting Summary

## Data Availability

Due to laws governing patient privacy, individual-level data is not available upon request.
