# Supplementary material for: Natural language processing system for rapid detection and intervention of mental health crisis chat messages
Source: NPJ Digit Med. 2023 Nov 21;6:213. doi: 10.1038/s41746-023-00951-3 (PMC10663535; doi:10.1038/s41746-023-00951-3)

# **Supplementary Information**

## Crisis Terms Filter

The crisis terms filter was constructed by sequentially combining three different sources of data: 1) a literature review of articles attempting to identify words and phrases that signal suicide risk, 2) a dataset of existing words and phrases that has been validated in similar studies on imminent harm classifiers and Twitter, and 3) true crisis messages from Cerebral that were incorrectly excluded by previous iterations of the terms filter. We iteratively reviewed these crisis messages and added words and phrases to the crisis terms list to prevent similar crisis messages from being excluded in the future. This review happened multiple times throughout the development of our crisis terms list, and continues to happen throughout the product life-cycle of CMD-1.

| Crisis | 800 mg | cut myself | Hate my life | really done | tears |
| --- | --- | --- | --- | --- | --- |
| Feel terrible | 11:11 | Slit | My time has come | exhausted | stop crying |
| Worst day of my life | Bathtub | Helpless | Kurt Cobain | given up | cry myself |
| Suicide | Ibuprofens | Help me | 13th reason | can’t take it | into a coma |
| Suicidal | Nightstand | Give up | Rope | fucking | haven't slept |
| Unsafe | Electrocute | Worthless | No longer want to live | freaking | insomnia |
| Hurt myself | Electrocution | Stop the pain | commit suicide | can't even | any sleep |
| Harm myself | Vampire | Hit by a bus | stab my throat | ugh | sleep deprivation |
| Kill | Bridge | Drown | slash my throat | crap | get out of bed |
| Kill myself | Railroad | Torture | cut my throat | pissed | no energy |
| Excedrin | Acetaminophen | Hadn't been born | slash my neck | angry | low energy |
| Die | Don't want to be here | Sleep forever | cut my forearm | wtf | can't wake up |
| Death | End it all | Can't this anymore | slash my forearm | shit | can't get up |
| Midnight | End my life | Failure | slit my forearm | I hate my | pistol |
| Syringe | Emergency | Hopeless | cut my wrists | fucked | rifle |
| Antifreeze | I just want this all to end | lack of hope | slash my wrists | bullshit | ar-15 |
| OD | Shoot | hopelessness | slit my wrists | lose my mind | magnum |
| Overdose | Relapse | No reason to live | take all these pills | disappoint | firearm |
| Train | Relapsed | Feeling trapped | take a handfull of these pills | Fail | dead |
| Tablets | Stab | Never wake up | swallow these pills | defeated | struggle |
| Hang | going to cut | Shotgun | at my throat | lonely | struggling |
| Noose | start cut | Weapon | attempt | loneliness | hospital |
| Revolver | wanna cut | Nothing left to live for | finish it | one care | can't go on |
| 800mg | Urge to cut | Lost everything | pull the trigger | wants me to die | off myself |
| suicidal thoughts | never been this close | gas myself | sharp | wants me to be dead | not living |
| suicidal urges | never been this bad | going downhill | grave | drunk | can't take it anymore |
| this urge | hang myself | gone downhill | passed away | drinking | lifeline |
| this is the only way | walk into traffic | poison myself | heaven | hangover | national hotline |
| I'm going to sleep forever | step into traffic | drink bleach | lost my | drink myself | negative thoughts |
| inject myself | no more hope | drink the whole bottle | lost a | abuse | I need counseling |
| leave everything behind | no point | Bleach | depressed | addiction | I need help |
| nobody can stop me | no hope | resist | cry | addicted | threat |
| you can't stop me | meet the reaper | temptation | depression | booze | threatened |
| It's too late | go into the great unknown | burn | despair | tablet | sadness |
| suicid | bleed out | start burn | unhappy | alcohol | lost all joy |
| so alone | fight is over | I'm done | freaking out | narcotics |  |
| go to sleep forever | fight this anymore | I’m just done | terrified | vodka |  |
| better off dead | gun | So done | nightmare | drugs |  |
| suicide plan | knife | end tonight | can’t breathe | drug |  |
| jump off | broken glass | ends tonight | freak out | pain killer |  |
| tired of life | blade | with life | need a break | pain killers |  |
| can't go on living like this | razorblade | end everything | I don't want to | cocaine |  |
| suicide note | razor | end it | falling apart | heroin |  |
| suicide letter | in my hands | self-harm | losing it | whiskey |  |
| not worth living | take my last breath | self harm | give in | lsd |  |
| suicide pact | blow my brains out | self-injury | no one cares | meth |  |
| take my life | it's too much | self injury | I have no one | shrooms |  |
| the worst it's ever been | suffocate | want to be alive | the end | weep |  |

SI Table 1: Message characteristics and patient demographic and clinical characteristics for the full prospective test set (n = 102,471).

| **Metric** | **Prospective test set** |
| --- | --- |
| **Message-level attributes** | |
| Total N distinct messages | 102,471 |
| N true crisis events (%) | 0.55% |
| Median N messages per patient (95% CI) | 2 (95% CI: 2-2) |
| Date range of messages included | 10/1/22 - 10/31/22 |
| Median N characters per message (95% CI) | 92 (95% CI: 91-92) |
| **Patient-level attributes** | |
| Total N distinct patients | 32,803 |
| Gender |  |
| Female | 22,258 (68%) |
| Male | 9,207 (28%) |
| Other | 1,338 (4.1%) |
| Age (years) at date of first message sent |  |
| 18-25 | 5,537 (17%) |
| 25-45 | 23,898 (73%) |
| 45-60 | 2,950 (9.0%) |
| >60 | 418 (1.3%) |
| Mental health diagnosis at the time of first message sent |  |
| Generalized anxiety disorder (+ other comorbidities) | 11,043 (34%) |
| Major depressive disorder (+ other comorbidities excluding GAD) | 9,233 (28%) |
| Bipolar disorder (+ other comorbidities excluding GAD and MDD) | 2,183 (6.7%) |
| Other | 9,006 (27%) |
| No diagnosis |  |
| Days from treatment initiation to first message sent |  |
| <2 weeks | 1,844 (5.6%) |
| 2-4 weeks | 26,010 (79%) |
| 4-8 weeks | 1,068 (3.3%) |
| 8-12 weeks | 1,750 (5.3%) |
| >12 weeks | 1,585 (4.8%) |
| Unknown | 546 (1.7%) |

SI Figure 1: Calibration curve, intercept, and slope for retrospective and prospective test set.


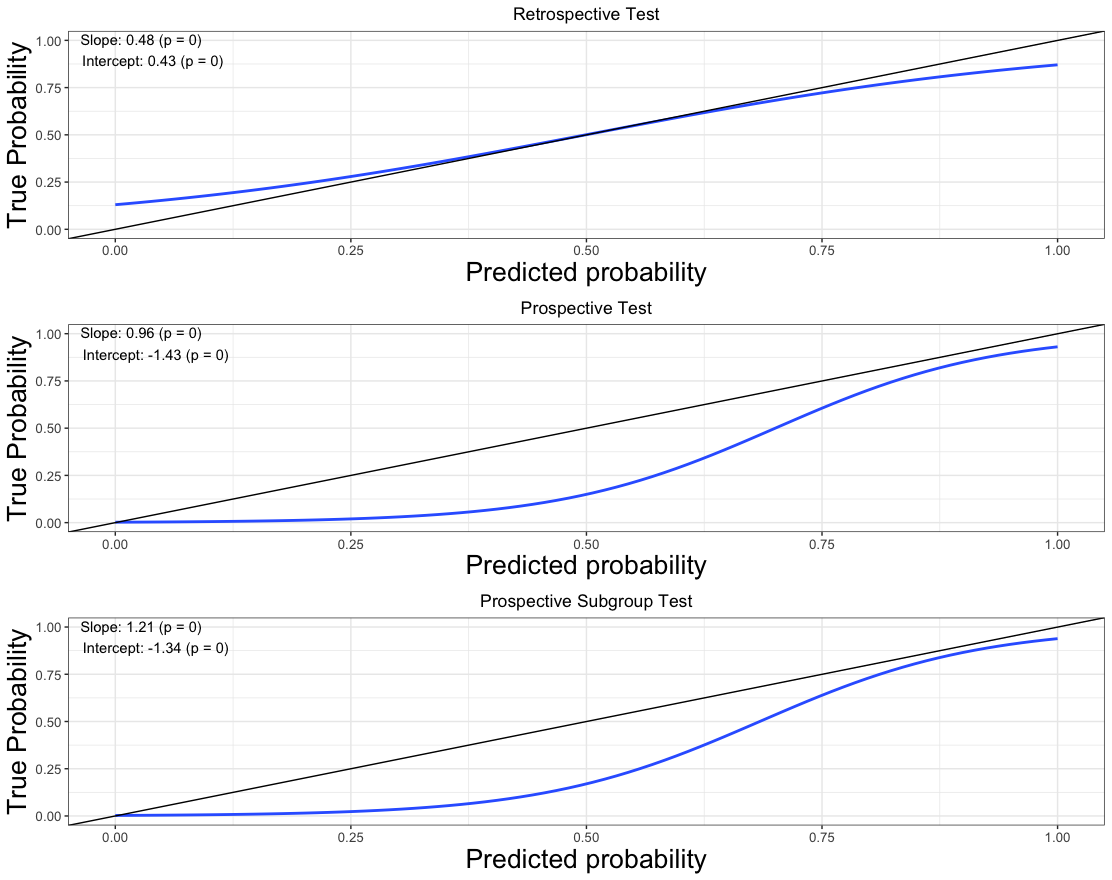


SI Figure 2: Calibration plots for retrospective and prospective test set showing the average predicted probability (red) and true event rate (blue) by decile of predicted probability (X-axis).


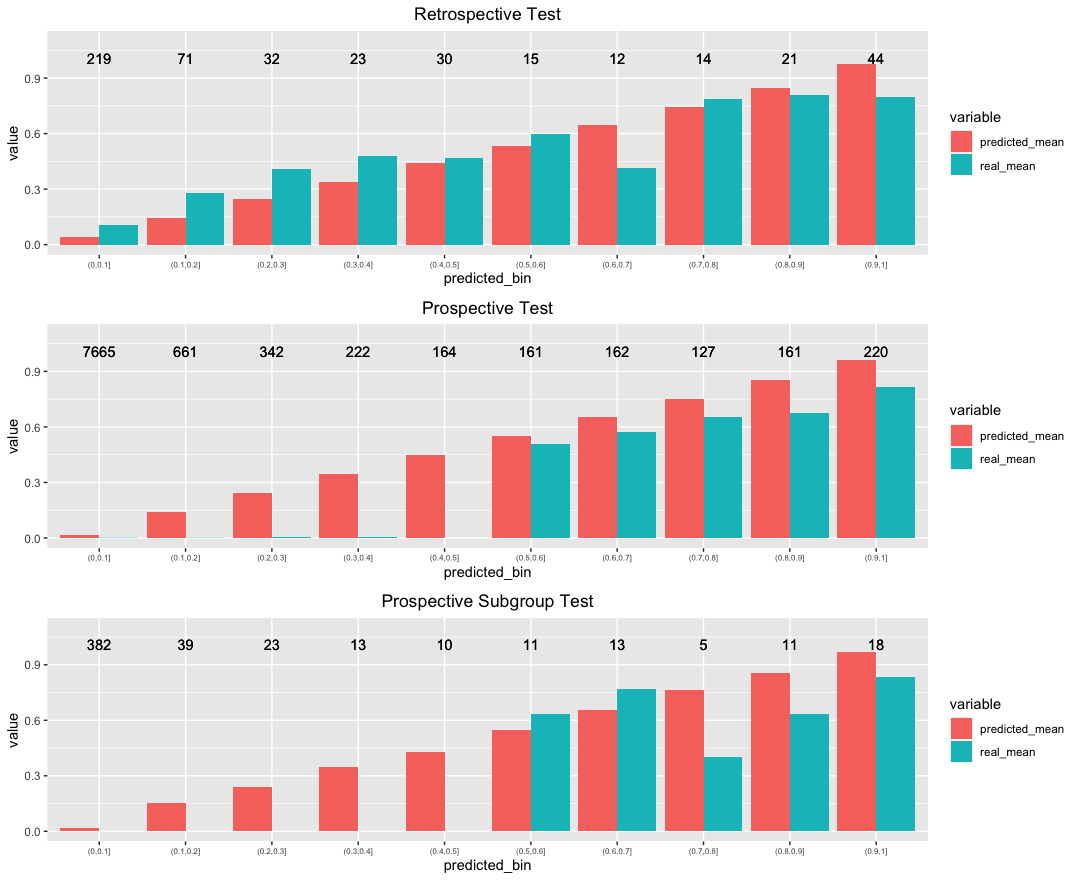


SI Table 2: Crisis terms filter performance metrics

| **Metric** | **Value** |
| --- | --- |
| Sensitivity | 0.993 (95% CI: 0.982-0.998) |
| Specificity | 0.910 (95% CI: 0.908-0.911) |
| PPV | 0.0567 (95% CI: 0.052-0.062) |
| NPV | 0.99996 (95% CI: 95% CI: 0.999-1.00) |

SI Table 3: Top 10 features selected from the model after training, along with coefficient from the L1-regularized logistic regression model. A positive coefficient indicates that the feature is associated with a crisis.

| **Feature** | **Coefficient** |
| --- | --- |
| suicidal | 141.19 |
| enough | 97.98 |
| thinking | 89.59 |
| i did | -78.46 |
| i could | 65.69 |
| being | -65.51 |
| to me | 64.47 |
| just wanted | -63.72 |
| suicide | 61.07 |
| where i | -60.98 |

SI Table 4: Summary of failure analysis reviewing false negatives.

| **False negative type** | **N** | **Reason(s) for incorrect prediction** | **Possible action to improve CMD-1 performance** |
| --- | --- | --- | --- |
| Did not pass through crisis term filter | 4 | Crisis term filter did not contain critical word/phrase (“ER”, “tired of being alive”, “not exist”, “not feeling okay”) | Add terms to crisis term filter |
| Passed through crisis term filter, model classified as non-crisis | 13 | Some messages contained mentions of medication-related crises, but the features learned by the model that were associated with medications had a negative association with the outcome | Retrain model on newly labeled data |

SI Figure 3: AUC Sensitivity Analysis results including AUC sensitivity analysis box plot and summary table reporting 1st and 3rd quartiles, median, minimum, and maximum, values of calculated AUC from 100 iterations of downsampling false negatives.


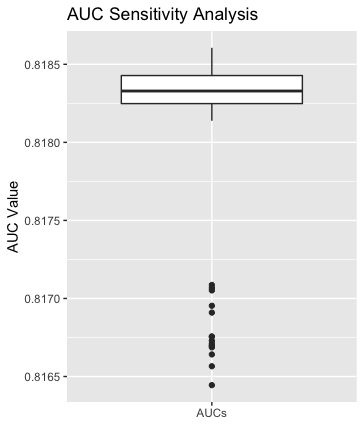


| **Min** | **1st Qu.** | **Median** | **3rd Qu.** | **Max** |
| --- | --- | --- | --- | --- |
| 0.8164 | 0.8182 | 0.8181 | 0.8184 | 0.8186 |

SI Table 5: Retrospective and Prospective test set true negative predictive probabilities reporting 1st and 3rd quartiles, median, minimum, and maximum, values

| **Dataset** | **Min** | **1st Qu.** | **Median** | **3rd Qu.** | **Max** |
| --- | --- | --- | --- | --- | --- |
| Retrospective test set | 0 | 0.026 | 0.079 | 0.17 | 1 |
| Prospective test set | 0 | 0.0027 | 0.019 | 0.069 | 1 |

SI Figure 4: Prospective test set Precision-Recall Curve plot with AUC = 0.753.


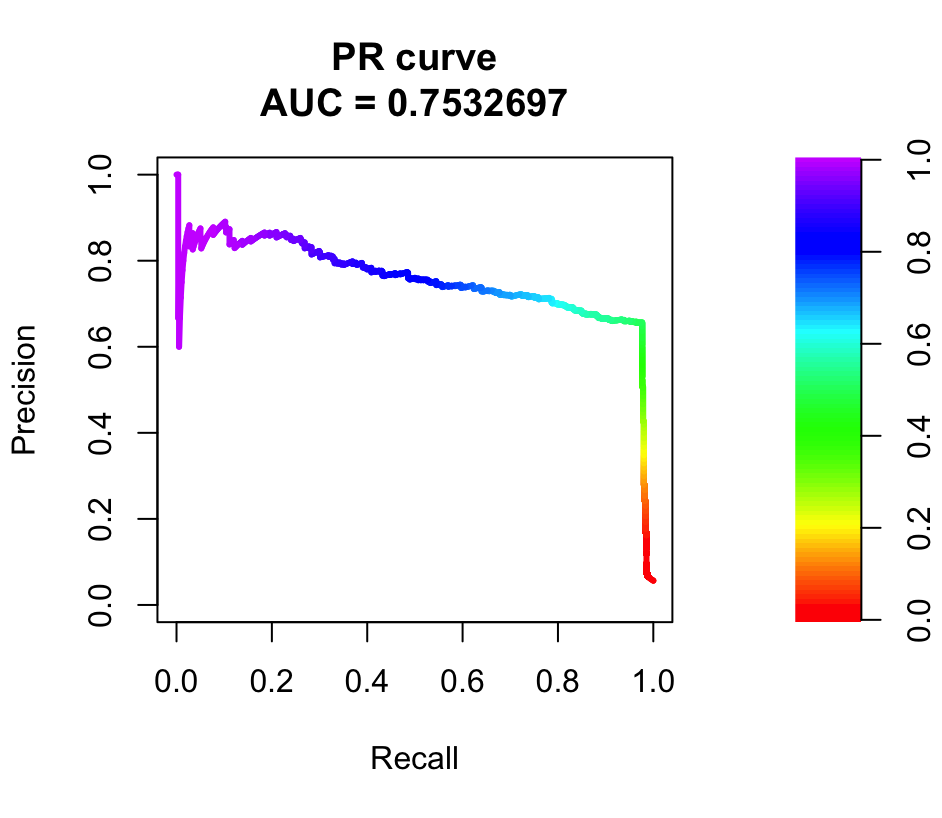

Supplement: Supplementary file 1 — Supplementary Information [file 41746_2023_951_MOESM1_ESM.docx]
